# Serum levels of androgens in acne & their role in acne severity

**DOI:** 10.12669/pjms.35.1.131

**Published:** 2019

**Authors:** Usma Iftikhar, Nakhshab Choudhry

**Affiliations:** 1 Dr. Usma Iftikhar, FCPS. Department of Dermatology, Mayo Hospital, King Edward Medical University, Lahore, Pakistan; 2 Dr. Nakhshab Choudhry, PhD. Professor & Chairman, Department of Biochemistry, King Edward Medical University, Lahore, Pakistan

**Keywords:** Acne vulgaris, Androgens, Hyperandrogenism, Acne Severity

## Abstract

**Objectives:**

To correlate acne severity with elevated androgen levels and to compare androgen levels between cases and controls.

**Methods:**

This case-control study was carried out in the Department of Dermatology, Mayo Hospital, Lahore from March 2016 – March 2017. Two hundred and seventy patients and eighty age and gender-matched controls were recruited after ethical approval and informed consent and categorized into mild, moderate and severe acne. Severity was correlated with serum Testosterone, Dihydrotestoststerone and Dihydroepiandrosterone Sulphate levels. Quantitative variables were expressed as median and percentiles, comparisons done by Mann-Whitney and correlations by Spearman correlation. P value of < 0.05 was considered statistically significant.

**Results:**

There were 142 (41%) males and 208 (59%) females. Ninety-Seven patients had mild, 108 moderate and 65 had severe disease. Median hormonal levels were 3.5ng/ml, 184pg/ml and 0.82ug/dl for Testosterone, Dihydrotestosterone and Dihydroepiandrosterone Sulphate respectively which differed significantly between cases and controls. There was no correlation with severity but the levels differed significantly between the different grades in case of Testosterone and DHEAS.

**Conclusion:**

Androgens are not directly correlated with acne severity, but affect acne severity as seen in difference between their levels in different grades of acne. Anti-androgens may be initiated early in acne resistant to conventional therapy.

## INTRODUCTION

Acne is one of the commonest dermatological disorders with a prevalence of 22%^1^ It is a disease of the pilosebaceous follicle with presence in 70-95% of dermatological patients.^2^ Endocrine mechanisms control the components of sebocyte function—namely lipid synthesis, proliferation and differentiation.^3^ Androgens upregulate sebaceous gland function by binding to the nuclear androgen receptors (ARs). Highest density of these has been demonstrated in sebaceous glands.

Testosterone (T) and dihydrotestosterone (DHT) are synthesized in the skin and bind to the androgen receptor.^4^ Clinical studies support the role of androgenic hormones in acne pathogenesis and cite the approximation of acne onset with surge of dihydroepiandrosterone sulphate (DHEA-S), acne in congenital adrenal hyperplasia (CAH), sudden onset acne, androgens administration leading to acne and improvement by androgen receptor blockers.

DHEA-S was found to be in the highest concentration in both sexes, which is converted by sebocytes and dermal papilla into testosterone and DHT. Androgen receptor (AR) malfunction is associated with acne.^5^ Patients with androgen excess, like Cushing’s syndrome, Polycystic Ovaries (PCOs) and CAH, can all have acne. However, there are conflicting studies regarding androgen levels in acne severity. A positive relationship was not found between grades of acne severity and hyperandrogenism.^6^ In some, raised DHEA-S levels were found.^7^ Androgens exhibit increased receptor sensitivity and 5α-reductase activity leading to high DHT levels.^8^

Acne may be associated with endocrinopathies, PCOs and ovarian tumours, if associated with hyperandrogenism. It is important to standardize the hormonal profile and control relapses in breakouts of acne. Treatment includes androgen receptor blocker, lowering androgen production by adrenals and ovaries.^9^

The objectives of this study were to correlate acne severity with elevated androgen levels and to compare androgen levels between cases and controls.

## METHODS

Two hundred and seventy acne patients and eighty age and gender matched controls presenting to the outpatient department of Mayo Hospital, Lahore were included. Patients were studied for hyperandrogenism. Patients having features of hyperandrogenism clinically (e.g. hirsutism) were excluded as well as those patients having a known cause of hyperandrogenism like PCOS or oral contraceptives in case of females or Cushing’s syndrome (as tested on Ultrasonography and fasting serum cortisol levels). Patients presenting for the first time were selected. After approval from the ethical committee and informed consent, after ruling out polycystic ovaries on ultrasound and after basal serum cortisol, fasting androgen levels were recorded. Levels were measured by ELISA using kits by Diasource Belgium and tests performed on Diamate 310. Levels of androgens were compared between cases and controls and correlated with acne severity. All data was entered into SPSS 20. Since the distribution was non-normal, quantitative data was expressed in median and percentiles. Non-parametric tests like Mann-Whitney were done for comparison between two groups. Correlation was measured by Spearman Correlation. A P value of < 0.05 was considered statistically significant.

## RESULTS

There were 142 (40.5%) males and 208 (59.4%) females. In cases, there were 155 (57%) females and 115 (43%) males, whereas in controls, there were 54 (67%) females and 26 (33%) males. In both males and females, the median age was 20 years. Median age of cases was 20 years and median age of controls was 21 years. Ninety-Seven (36%) of patients had mild disease, 108 (40%) had moderate disease and 65 (24%) had severe disease.

In the comparison of hormonal levels between cases and controls, there was a statistically significant difference (Table-I) tested by Mann-Whitney Test.

**Table-I:** Comparison of Androgenic Hormones between Cases and Controls.

Hormones		N	Median	IR	P
Testosterone (ng/ml)	Case	270	0.82	(0.56,5.77)	<.001
	Control	80	0.45	(0.36,4.99)	
DHT (pg/ml)	Case	270	350.24	(280,545.7)	<.001
	Control	80	280	(215,375.45)	
DHEAS (µg/dl)	Case	270	1.84	(1.27,2.55)	<.001
	Control	80	0.72	(0.52,2.42)	

N= Number of Patients, SD= Standard Deviation, IR = Interquartile Range, P = Significance DHT = Dihydrotestosterone, DHEAS = Dihydroepiandrosterone Sulphate

In the correlation of serum levels of hormones with severity, there was no significant correlation (Table-II).

**Table-II:** Correlation between Levels of Hormones & Severity (Spearman Correlation)

Correlation	N	r	P
Testosterone VS Severity	270	0.077	0.205
DHT VS Severity	270	0.085	0.165
DHEAS VS Severity	270	0.104	0.088

**. Correlation is significant at 0.01 level (2 tailed) N= Number of Patients, SD= Standard Deviation, P = Significance DHT = Dihydrotestosterone, DHEAS = Dihydroepiandrosterone Sulphate P = Significance, r = Correlation Co-efficient

In the comparison of hormonal levels between different grades of severity, there was a significant difference in case of Testosterone and DHEAS (Table III). Although there is a statistically significant difference in the values of hormones, there is no proportional correlation with severity (Table-II).

**Table-III:** Comparison of Level of Androgenic Hormones between different grades of Severity. Since the data is non-parametric, median values are considered and mean/SD are obliterated.

	Severity	N	Median	IR	x^2^	P
Testosterone (ng/ml)	Mild	97	0.65	(0.51,5.2)	14.04	0.001
	Moderate	108	1.4	(245.57,370.27)		
	Severe	65	2.48	(238.75,413.50)		
DHT (pg/ml)	Mild	97	404.97	(271.45,493.80)	2.99	0.223
	Moderate	108	367	(283,600.50)		
	Severe	65	359	(289,534.6)		
DHEAS (µg/ml)	Mild	97	1.7	(1.20,2.40)	6.37	0.041
	Moderate	108	1.98	(1.39,2.69)		
	Severe	65	1.8	(1.40,2.43)		

N= Number of Patients, SD= Standard Deviation, IR = Interquartile Range, P = Significance DHT = Dihydrotestosterone, DHEAS = Dihydroepiandrosterone Sulphate, P = Significance

## DISCUSSION

In this study, the median age was 20 years. In the study by Kiyani et al.^10^, the mean age was 21.48 ± 4.73 years. In the study by Rahaman et al in India^11^, the mean age of patients was 19.83 years, similar to this study. In the study by Saleh et al.^12^ the mean age was 21.17 ± 4.21 years, also similar to this study. This coincides with the sebaceous activity at this age group under influence of androgens.^4^

Thirty six percent patients had mild, 40% moderate and 24% had severe disease. The highest proportion comprised of patients with mild disease. This is similar to the study by Kaiyani et al.^10^ This can be attributed to the inclusion criteria of selecting cases presenting for the first time.

The levels of androgenic hormones are given in Table-I. In the study by Da Cunha et al.^13^, 54% of the patients had hyperandrogenism and DHEAS levels were most frequently elevated. In the study by Borgia et al.^14^, there was elevation of at least one androgen.

Marynick SP et al.^15^ in their study found that affected patients had increased testosterone and DHEA-S levels. In the study by Slayden M et al.^16^, acne was related to hyperandrogenism irrespective of the age of presentation. Sixty-three percent of patients had at least one androgen level above 95% of normal. The levels of Total T were elevated in 38% of patients in 12-18 years of age and 5% in the 19 - 43 years age group, DHEAS levels were elevated (>7.6 µmol/L) in 75% cases in 12-18 years and in 36% in 19 -43 year age group. In the study by Uysal et al.^8^, 55% of women with acne had elevation of at least one androgen.

In this study, there was a significant difference in androgen levels between cases and controls (Table-I). In another study^17^, there was no difference between cases and controls in case of Total Testosterone, Free Testosterone and DHT except DHEAS (P value <0.05). In the study by Vexaiu et al.^18^, there was a significant difference in levels of androgenic hormones between cases and controls, namely Testosterone 0.5 ng/ml versus 0.35 ng/ml, Andostenedione 2.2 ng/dl versus 1.3 ng/dl, DHEAS 3.5 µg/dl versus 2.5 µg/dl in cases and controls respectively. In the study in Bangladesh,^19^ 10% acne patients had testosterone above normal levels.

There was no significant correlation of androgens with severity (Table-II) with Spearman Correlation. In the study by Kaiyani et al.^10^, no statistically significant relationship was demonstrated between testosterone levels and acne severity. In the study in Iran by Zandi S et al.^20^, comparison of hormones between patients having PCOS and non-PCOs, levels of testosterone and dihydroepiandrosterone were not statistically significant. In the study by Aizawa et al.^17^, there was no correlation between androgen levels and severity.

Adityan B^21^ et al in their study found that there was no association between acne severity and hyperandrogenism. In the study by Borgia et al.^14^, although hyperandrogenemia was present, no correlation was found with severity. In the study by Henze et al.^22^, more than 30% of patients had elevated testosterone or DHEAS levels. However, large numbers with severe acne had normal androgens. This could be attributed to the fact that neither testosterone, nor DHEAS have strong androgenic potential in the pilosebaceous unit. Rather local enzymes are responsible for reduction of testosterone to 5α-dihydrotestosterone. Severe virilization could be caused by the overactivity of either 5-α reductase^22^ or androgen receptor.

In this study, comparison of androgens between the three grades of acne revealed significant difference in Testosterone and DHEAS levels (Table-III), but not in DHT. In the study by Cibula et al.^6^, no positive correlation was found between acne severity and clinical/ laboratory values of androgenism. Women with increased severity had lower free testosterone. Contrary to this, in the study by Saleh et al.^12^, levels of testosterone, dihydroepiandrosterone and androstenedione were significantly raised in severe acne as compared to moderate (p = <0.005) and mild acne (p = 0.001).

Lucky AW et al.^23^ in their study found that females with severe comedonal acne had significantly higher DHEA-S and testosterone levels. In the study by Seraifi et al.^24^, only DHEA-S was raised in women with acne but not hyperandrogenism, similar to this study. Similarly, in India^25^ although higher testosterone levels were found in cases compared to controls, statistically significant difference was observed in moderate and severe acne only. Thus, the role of androgens in acne pathogenesis can be observed by the difference in their serum levels, especially in the case of Testosterone and DHEAS.^25^

## CONCLUSION

Although androgenic hormones may be raised in the acne patients as compared to controls, only Testosterone and DHEA-S levels serve as markers of acne severity. Any therapy targeted towards acne and its severity can, therefore, be directed towards the ovarian androgen production, or blocking of androgen receptors in the pilosebaceous unit. Thus hormonal therapy can be an adjuvant to early treatment in acne.

### Authors’ Contribution

**UI:** Conceived, designed, and performed statistical analysis.

**NC:** Helped in analysis and interpretation of data, final approval of the manuscript.
